# Antiviral Activity of Microbial Metabolites Monensin and Brefeldin A Against Toscana Virus: In Vitro Evaluation and Mechanistic Insights

**DOI:** 10.3390/v18030287

**Published:** 2026-02-27

**Authors:** Laura Di Clemente, Carla Zannella, Francesca Palma, Marina Acunzo, Rosa Giugliano, Annalisa Chianese, Floriana Bonura, Simona De Grazia, Giovanni M. Giammanco, Anna De Filippis, Massimiliano Galdiero

**Affiliations:** 1 Department of Woman, Child and General and Specialized Surgery, University of Campania Luigi Vanvitelli, 80138 Naples, Italy; laura.diclemente@unicampania.it (L.D.C.); carla.zannella@unicampania.it (C.Z.); francesca.palma@unicampania.it (F.P.); marina.acunzo@unicampania.it (M.A.); rosa.giugliano@unicampania.it (R.G.); annalisa.chianese@unicampania.it (A.C.); anna.defilippis@unicampania.it (A.D.F.); 2 Department of Veterinary Medicine and Animal Production, University of Naples Federico II, 80137 Naples, Italy; 3 Department of Life Sciences, Health and Health Professions, Link Campus University, 00165 Rome, Italy; 4 Department of Health Promotion, Mother and Child Care, Internal Medicine and Medical Specialties “G. D’Alessandro”, University of Palermo, 90127 Palermo, Italy; floriana.bonura@unipa.it (F.B.); simona.degrazia@unipa.it (S.D.G.); giovanni.giammanco@unipa.it (G.M.G.); 5 UOC Virology and Microbiology, University Hospital “Luigi Vanvitelli”, 80138 Naples, Italy

**Keywords:** Toscana virus, phlebovirus, arboviral infection, microbial secondary metabolites, monensin, brefeldin A, antiviral activity, Golgi stress response, TFE3

## Abstract

Toscana virus (TOSV), a negative-sense RNA phlebovirus transmitted by *Phlebotomus* sandflies and endemic in Mediterranean regions, is an emerging pathogen capable of causing diseases ranging from mild febrile illness to severe central nervous system involvement. With no licensed vaccines or specific antiviral therapies available, the identification of novel therapeutic approaches is urgently needed. Microbial secondary metabolites have recently attracted attention for their broad-spectrum antiviral activities. Among them, monensin and brefeldin A have shown antiviral efficacy against a variety of viruses, often by disrupting viral protein trafficking and inducing Golgi-associated stress responses. However, their potential activity in the context of TOSV infection has not yet been explored. This study aimed to evaluate the in vitro antiviral activity of monensin and brefeldin A against TOSV and to gain mechanistic insights into their effects at the cellular level. Vero cells were infected with TOSV and treated with monensin (1.5–25 µM) or brefeldin A (10.9–175 nM) at different time points (4, 6, 12, 24 h). Cytotoxicity was assessed using MTT and hemolysis assays. Antiviral activity was measured via plaque reduction assays and quantitative real-time PCR targeting the viral *L* gene. Western blot analysis was performed to assess TFE3 expression, a transcription factor associated with the Golgi stress response. Monensin exhibited rapid antiviral activity, achieving IC_50_ values of 2.7 µM and 2.5 µM at 4 and 6 h post-treatment, respectively, with dose-dependent suppression of viral *L* gene expression. Brefeldin A displayed a delayed effect, with maximal inhibition after 12 h (IC_50_ = 66.9 nM). Monensin treatment induced a concentration-dependent upregulation of TFE3, while brefeldin A caused only a modest increase, suggesting differential activation of the Golgi stress response during TOSV infection. These findings support the potential of microbial metabolites as therapeutic candidates for emerging arboviral infections in the Mediterranean region.

## 1. Introduction

Toscana virus (TOSV) is a tripartite, negative-sense, single-stranded RNA virus belonging to the species *Phlebovirus toscanaense*, within the genus *Phlebovirus* of the family *Phenuiviridae* [1,2]. It is an emerging arthropod-borne virus primarily transmitted by *Phlebotomus* sandflies, which are widely distributed throughout Mediterranean countries, including Italy, Spain, Portugal, France, Turkey, Croatia, Greece, Algeria, and Tunisia [3,4]. First isolated in Tuscany, Italy, in 1971, TOSV infection in humans ranges from asymptomatic or mild febrile illness to severe central nervous system (CNS) manifestations, including meningitis, meningoencephalitis, with rare cases progressing to life-threatening complications or death [5]. Between 1985 and 2023, 1381 cases were reported across 12 countries, most of them occurring in Italy, reflecting a rising incidence of infection [6].

This epidemiological scenario underscores the urgent need to develop specific antiviral therapies against TOSV, as no effective treatments are currently available. In parallel, the escalating threat of antimicrobial resistance has driven a concerted search for novel and sustainable bioactive compounds capable of inhibiting emerging pathogens. Within this context, microbial derivatives have attracted increasing attention for their broad-spectrum antiviral properties [7]. Microorganisms possess an exceptional capacity to synthesize diverse secondary metabolites, many of which exhibit antiviral activity coupled with low cytotoxicity.

Microbial secondary metabolites can be classified based on their chemical structure, biosynthetic origin, or biological function. Chemically, they include alkaloids, polyketides, terpenoids, non-ribosomal peptides, and polyphenolic compounds [8,9]. Biosynthetically, they are produced by distinct enzymatic systems; for example, polyketide synthases generate polyketides [10], whereas alkaloids typically arise from amino acid precursors [11]. Functionally, these metabolites serve as direct therapeutics, surface disinfectants, and immune-enhancing supplements [12,13]. Among these, bioactive polysaccharides and biosurfactants represent notable classes [14]. For instance, fungal polysaccharides such as lentinan from *Lentinus edodes* exhibit activity against infectious hematopoietic necrosis virus [15], while polysaccharides from *Inonotus obliquus* inhibit Newcastle disease virus [16]. Similarly, bacterial polysaccharides such as Nostoflan from *Nostoc flagelliforme* demonstrate antiviral activity against herpes simplex virus type 1 (HSV-1) and type 2 (HSV-2), human cytomegalovirus (HCMV), and influenza A virus [17]. Microbial biosurfactants, including rhamnolipids, also exhibit antibacterial, antifungal, and antibiofilm effects; for example, rhamnolipids produced by *Pseudomonas gessardii* display antiviral activity against HSV-1, HSV-2, and coronaviruses [18].

In the present study, we focused on two microbial secondary metabolites: monensin and brefeldin A. Monensin is a polyketide produced by the bacterium *Streptomyces cinnamonensis* and is widely used as an ionophore antibiotic in ruminant feed to improve efficiency and control specific infections [19]. During the 1980s, it was extensively investigated as an antiviral compound and demonstrated activity against multiple viruses, including HSV-1, HSV-2, HCMV [20,21,22], Uukuniemi virus [23], and Semliki Forest virus [24]. More recently, monensin regained interest due to its reported antiviral effects against several coronaviruses, including SARS-CoV-2 and the human coronaviruses HCoV-229E and HCoV-OC43 [25]. Its antiviral activity is primarily mediated through the inhibition of viral protein transport from the Golgi apparatus to the cell surface, resulting in intracellular virion accumulation and reduced release of infectious particles [26]. Monensin also activates the transcription factor TFE3, a key regulator of the Golgi stress response that promotes the expression of genes such as *GOLGA2*, *GOLGB1*, and *ACBD3*, thereby contributing to Golgi restoration [27]. This cellular response is particularly relevant during viral infections, given the critical role of the Golgi in the assembly, maturation, and release of newly formed infectious viral particles [28]. Brefeldin A is a lactone-derived fungal metabolite originally isolated from *Eupenicillium brefeldianum*. It has demonstrated potent anticancer activity across several tumor types, including lung, colorectal, ovarian, breast, prostate, melanoma, and CNS cancers, highlighting its potential as a valuable therapeutic molecule [29]. In addition to its anticancer properties, brefeldin A has been widely studied for its antiviral activity. Its mechanism involves inhibition of protein trafficking from the endoplasmic reticulum to the Golgi apparatus, leading to Golgi disassembly, a process essential for the replication, assembly, and maturation of many viruses. Its antiviral activity has recently been explored against flaviviruses such as dengue virus, Zika virus, and Japanese encephalitis virus [30].

This study aimed to evaluate the in vitro antiviral activity of monensin and brefeldin A against TOSV and to gain mechanistic insights into their effects at the cellular level.

## 2. Materials and Methods

### 2.1. Cell and Virus Growth

Vero cells (ATCC CRL-1587) were grown at 37 °C with 5% CO_2_ in Dulbecco’s Modified Eagle Medium (DMEM) containing 4.5 g/L glucose (Microtech, Naples, Italy) and supplemented with 100× antibiotic solution (100 IU/mL penicillin and 100 μg/mL streptomycin; Himedia, Naples, Italy), and 10% Fetal Bovine Serum (FBS, Microtech). TOSV (strain Phl.3, ISS, European Virus Archive, Marseille, France) was kindly provided by Professor G. Giammanco and Prof. Simona De Grazia (University of Palermo, Italy) and propagated on Vero cells. The virus stock had a titer of 5.9 × 10^10^ PFU/mL and was titrated using a plaque-forming assay on Vero cells. All experiments involving TOSV were performed under Biosafety Level 2 (BSL-2) conditions, in compliance with institutional biosafety regulations.

### 2.2. Compounds Preparation

**Figure 1:**
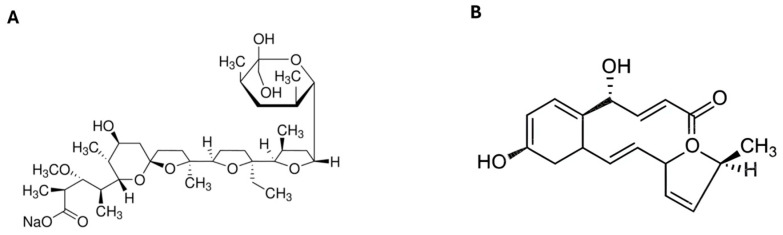
Chemical structures of monensin and brefeldin A. (**A**) Structure of monensin. (**B**) Structure of brefeldin A. Both structures were obtained from the manufacturers’ datasheets.

Monensin sodium salt (M5273, Sigma-Aldrich^®^, St. Louis, MO, USA) was dissolved in ethanol to a final concentration of 10 mM, while brefeldin A (203729, Sigma-Aldrich^®^) was prepared in methanol at a concentration of 1 mg/mL, according to the manufacturer’s instructions. Their chemical structures are reported in Figure 1.

### 2.3. Cytotoxicity of Monensin and Brefeldin A

The in vitro cytotoxicity of monensin and brefeldin A was evaluated using the 3-(4,5-dimethylthiazol-2-yl)-2,5-diphenyltetrazolium bromide (MTT) assay on Vero cells. Cells were seeded at a density of 2 × 10^4^ cells per well in a 96-well plate and incubated for 24 h (h). The following day, serial dilutions of monensin (1.56–100 µM) and brefeldin A (10.9–700 nM) were added to the cell monolayers and incubated at 37 °C for 6 and 24 h. Cytotoxicity was assessed by adding MTT solution (Sigma-Aldrich) to each well. After 4 h of incubation, the medium was removed, and formazan crystals were solubilized with 100% dimethyl sulfoxide (DMSO). Cell viability was determined by measuring absorbance at 570 nm using a microplate reader. The negative control (CTRL−, toxicity control) consisted of cells treated with DMSO, while the positive control (CTRL+, viable cells) included untreated cells. The percentage of cell viability was calculated using the formula:

(1) % cell viability=[(Absorbance sample )/(Absorbance untreated cells)]×100

To further evaluate the potential lytic effect of monensin and brefeldin A on cells, hemolytic activity was assessed using fresh human erythrocytes obtained from healthy anonymous donors. Briefly, blood was centrifuged, and erythrocytes were washed three times with 150 mM NaCl solution. Cells were then diluted 1:50 in phosphate-buffered saline (PBS, Sigma-Aldrich, pH 7.4), and 190 μL of the red blood cell suspension was added. Monensin and brefeldin A were applied at the same concentrations used in the MTT assay. Untreated erythrocytes and a 1% Triton X-100 solution served as CTRL− and CTRL+, respectively. Plates were incubated under orbital shaking at 37 °C for 1 h, then centrifuged at 500 rpm for 5 min. Hemoglobin release was quantified by measuring the optical density of the supernatant at 540 nm, and the hemolysis percentage was calculated using the formula:

(2) % hemolysis=[(Absorbance sample)/(Absorbance Triton)]×100

### 2.4. Plaque Reduction Assays

The antiviral activity of monensin and brefeldin A was evaluated using plaque reduction assays in a post-infection treatment scheme. Vero cells were seeded in 24-well plates at a density of 1.2 × 10^5^ cells per well and incubated overnight at 37 °C. Cells were then infected with TOSV at a multiplicity of infection (MOI) of 0.01 for 1 h, washed with PBS to remove unbound virus, and subsequently treated with monensin (1.5–25 µM) or brefeldin A (10.9–175 nM) [31,32]. Treatments were applied for 4, 6, 12, and 24 h at 37 °C. After incubation, cell monolayers were washed with PBS and overlaid with 3% carboxymethylcellulose (CMC) in culture medium. Following 24 h of incubation, cells were fixed with 4% formaldehyde and stained with 0.5% crystal violet. The positive control (CTRL+) consisted of bovine lactoferrin (1 mg/mL), while untreated infected cells served as the negative control (CTRL−) [33]. Viral plaques were counted, and the percentage of viral inhibition was calculated using the formula:

(3) $$\%\;\mathrm{viral}\;\mathrm{inhibition}=(1-\frac{Plaques\;counted\;in\;treated\;cells}{Plaques\;counted\;in\;untreated\;infected\;cells})\times 100$$

### 2.5. Real-Time Quantitative PCR (qRT-PCR)

Vero cells (1.2 × 10^5^ cells/well) were seeded in 24-well plates and incubated at 37 °C for 24 h. Infection was performed as described above. After viral adsorption, the cells were washed with PBS to remove unbound virus and then exposed to the compounds. Following compound exposure, the treatment medium was replaced with fresh culture medium after a single wash, and the cells were incubated for an additional 24 h at 37 °C. Both the cellular fraction and the extracellular supernatant were collected. Total RNA was extracted using TRIzol reagent (Thermo Fisher Scientific, Waltham, MA, USA), subjected to freeze–thaw cycles to promote efficient RNA release [34], and reverse-transcribed into cDNA using the 5× All-In-One RT MasterMix Kit (Applied Biological Materials, Richmond, BC, Canada) [35]. Quantitative real-time PCR (qRT-PCR) was performed to detect the expression of the viral L segment of TOSV. Cycle threshold (Ct) values of the target gene in infected, treated cells were compared to those in infected, untreated controls and normalized to the housekeeping gene glyceraldehyde 3-phosphate dehydrogenase (GAPDH). The primer sequences used were as follows: GAPDH (forward: 5′-CCTTTCATTGAGCTCCAT-3′; reverse: 5′-CGTACATGGGAGCGTC-3′), TOSV *L* gene (forward: 5′-GCATGGTGTGAGGACATGCAG-3′; reverse: 5′-TCTTGGTCTTTTATCTTTGC-3′). Relative quantification of viral RNA levels was calculated using the 2^−∆∆Ct^ method.

### 2.6. Western Blot

To investigate the effects of monensin and brefeldin A on TFE3 protein expression, Western blot analysis was performed. A post-treatment assay was conducted as previously described. Proteins from the cellular fraction were collected using RIPA buffer after 4 h of exposure to monensin (6.25, 12.5, and 25 µM) and after 12 h of exposure to brefeldin A (87.5 and 175 nM) [36]. Protein concentrations were determined using a spectrophotometer, and 20 μg of protein per sample were separated by 10% sodium dodecyl sulfate-polyacrylamide gel electrophoresis (SDS-PAGE) and transferred to nitrocellulose membranes. Membranes were blocked with 3% bovine serum albumin (Bio-Rad, Hercules, CA, USA) at room temperature for 1 h, followed by overnight incubation at 4 °C with primary antibodies against GAPDH (E-AB-40337, 1:1000, Elabscience, Houston, TX, USA) and TFE3 (14480-1-AP, 1:1000, Proteintech, Rosemont, IL, USA). After washing with PBS with Tween ^®^ detergent, membranes were incubated with a rabbit secondary antibody (E-AB-1003, 1:10,000, Elabscience) at room temperature for 1 h. Proteins were visualized using the ECL Western blotting detection system (Bio-Rad) and quantified with ImageLab 6.1 software. Bands were normalized to GAPDH [37].

### 2.7. Statistics Analysis

All experiments were performed in triplicate, and results are expressed as mean ± standard deviation (SD). Statistical analyses were conducted using GraphPad Prism (version 8.0.1). One-way analysis of variance (ANOVA), followed by Dunnett’s multiple comparisons test, was used to assess statistical significance. A *p*-value ≤ 0.05 was considered statistically significant. The 50% cytotoxic concentration (CC_50_) and the 50% inhibitory concentration (IC_50_) were determined by nonlinear regression analysis from dose–response curves generated from at least three independent experiments. The selectivity index (SI) was calculated considering the data related to efficacy and cytotoxicity (CC_50_/IC_50_).

## 3. Results

### 3.1. Cytotoxicity of Monensin and Brefeldin A

**Figure 2:**
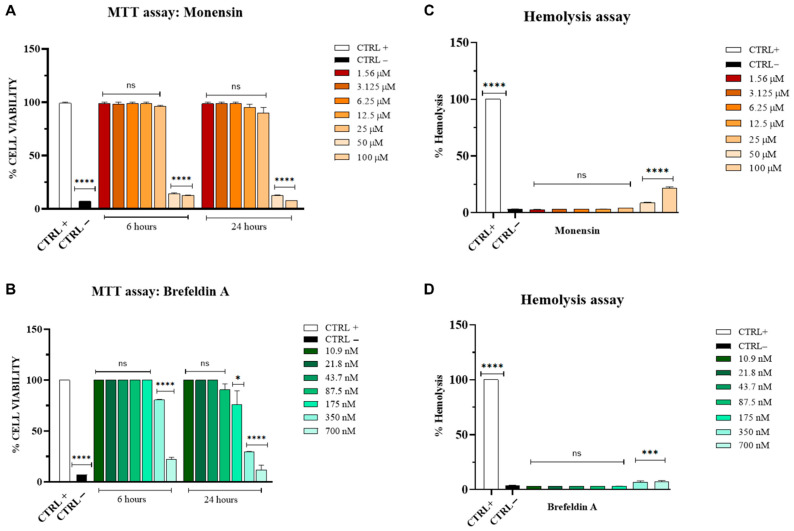
Assessment of cytotoxicity and hemolytic effects of monensin and brefeldin A. (**A**) Cell viability following monensin exposure, assessed by MTT assay. (**B**) Cell viability following brefeldin A exposure, assessed by MTT assay. (**C**) Hemolytic activity of human erythrocytes after monensin treatment. (**D**) Hemolytic activity of human erythrocytes after brefeldin A treatment. The positive control (CTRL+) consisted of untreated cells for the MTT assay and 1% Triton X-100 for the hemolysis assay, while the negative control (CTRL–) consisted of 100% DMSO for the MTT assay and untreated erythrocytes for the hemolysis assay. Statistical analysis was performed using one-way ANOVA followed by Dunnett’s multiple comparisons test. **** *p* < 0.0001; *** *p* < 0.01; * *p* = 0.0424; ns = not significant.

To determine the safety profile of monensin and brefeldin A, we evaluated their cytotoxic effects in Vero cells using the MTT assay (Figure 2A) and assessed hemolytic activity in human erythrocytes (Figure 2B).

Both compounds exhibited dose-dependent cytotoxic effects, particularly at the highest concentrations tested, i.e.,100 µM and 50 µM for monensin, and 700 nM for brefeldin A, after 6 h of exposure. After 24 h of treatment, cytotoxicity remained evident only at the highest concentrations, whereas lower concentrations did not induce detectable cell damage. In the control conditions, cell viability was 100% for the positive control (untreated cells) and 7% for the negative control, represented by 100% DMSO. The calculated CC_50_ values for monensin and brefeldin A are indicated in Table 1.

Results from the hemolysis assay (Figure 2C,D) were consistent with the MTT data, showing hemolytic activity of 21.5% and 8.6% for monensin at 100 µM and 50 µM, respectively, and <10% for brefeldin A at 700 nM.

### 3.2. Antiviral Activity of Monensin Against TOSV

**Figure 3:**
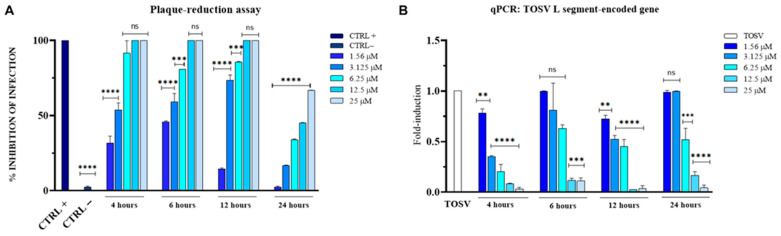
Antiviral activity of monensin against TOSV infection in Vero cells. (**A**) Evaluation of antiviral activity by plaque assay under post-treatment conditions at 4, 6, 12, and 24 h of treatment. Bovine lactoferrin was used as a positive control (CTRL+), and infected untreated cells served as a negative control (CTRL−). (**B**) Quantitative real-time PCR analysis of *L* segment-encoded gene expression. Statistical analysis was performed using one-way ANOVA followed by Dunnett’s multiple comparisons test. **** *p* < 0.0001; *** 0.0001 < *p* < 0.0003; ** *p* < 0.01; ns = not significant.

To assess the ability of monensin to inhibit viral replication, we evaluated its antiviral efficacy against TOSV using a post-treatment plaque reduction assay and qPCR. Briefly, infected cells were treated with the compound for 4, 6, 12, and 24 h, with the strongest inhibitory activity observed at the earliest time points (Figure 3A). In the control conditions, the positive control (bovine lactoferrin) showed 100% inhibition of viral infection, whereas no inhibition was observed in the negative control (infected untreated cells).

To further evaluate the impact of monensin on viral replication, we quantified the expression of the TOSV *L* segment, which encodes the viral RNA-dependent RNA polymerase, using qPCR (Figure 3B). Monensin induced a clear dose-dependent reduction in viral RNA levels. Notably, the highest concentrations tested (25 and 12.5 µM) resulted in a significant decrease in *L* gene expression compared with the untreated control at all analyzed time points. Table 2 summarizes the IC_50_ values of monensin at the different time points analyzed.

### 3.3. Antiviral Activity of Brefeldin A Against TOSV

**Figure 4:**
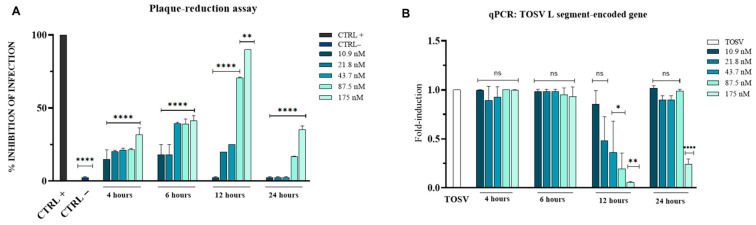
Antiviral activity of brefeldin A against TOSV infection in Vero cells. (**A**) Antiviral effect evaluated by plaque assay at post-treatment time points of 4, 6, 12, and 24 h. Bovine lactoferrin was used as a positive control (CTRL+), and infected untreated cells served as a negative control (CTRL−). (**B**) Quantitative real-time PCR analysis of *L* gene expression. One-way ANOVA with Dunnett’s multiple comparisons test was performed. **** *p* < 0.0001; ** *p* < 0.01; * *p* < 0.05; ns = not significant.

To investigate whether brefeldin A could inhibit TOSV replication, we assessed its antiviral activity under post-treatment conditions using plaque reduction and qPCR assays in the Vero cell line (Figure 4).

Brefeldin A exhibited its strongest inhibitory effect after 12 h of exposure, with an IC_50_ value of 66.9 nM, whereas no significant inhibition was observed at the other time points tested (Figure 4A). In the control groups, the positive control resulted in complete viral inhibition, while the negative control had no inhibitory effect. These results were confirmed by qPCR analysis of viral *L* segment expression (Figure 4B). No suppression of viral gene expression was detected after 4 or 6 h of exposure. Table 3 indicates the IC_50_ values of brefeldin A at the different time points analyzed.

### 3.4. Western Blot Analysis of TFE3

**Figure 5:**
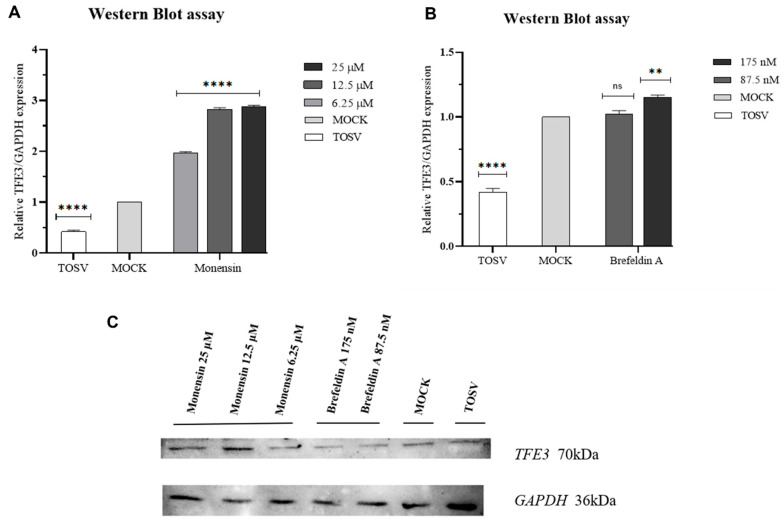
Western blot analysis of TFE3 protein expression in Vero cells following TOSV infection. (**A**) Cells were treated with 6.25, 12.5, and 25 μM of monensin for 4 h. (**B**) Cells were treated with 87.5 and 175 nM of brefeldin A for 12 h. (**C**) Representative blot images. Protein levels were normalized to GAPDH and expressed relative to mock-treated, uninfected controls. Statistical analysis was performed using one-way ANOVA, followed by Dunnett’s multiple comparisons test. Statistical significance is related to mock-treated, uninfected controls: **** *p* < 0.0001; ** *p* = 0.0052; ns = not significant.

To investigate the impact of monensin and brefeldin A on the Golgi stress response during TOSV infection, TFE3 protein expression was assessed by Western blot (Figure 5).

Infection with TOSV in the absence of any compound led to a 58% reduction in TFE3 protein levels relative to mock-treated, uninfected controls. Monensin induced a concentration-dependent upregulation of TFE3 after 4 h of treatment, with increased expression observed at 6.25, 12.5, and 25 µM. In contrast, exposure to 175 nM brefeldin A for 12 h resulted in a modest 15% increase in TFE3 protein expression compared with mock-treated, uninfected controls.

## 4. Discussion

Arboviral diseases are becoming an increasingly significant global health concern due to converging factors such as climate change, globalization, and urban expansion, all of which facilitate the spread of arthropod vectors and the pathogens they transmit [38]. Among these, TOSV infections have shown a marked rise, positioning this phlebovirus as an emerging public health threat across Mediterranean regions [39]. Despite its clinical relevance, ranging from febrile illness to neuroinvasive disease, no licensed vaccines or virus-specific antiviral treatments are currently available [33]. Therefore, identifying molecules that can interfere with TOSV replication is a pressing need. Microbial metabolites represent a valuable reservoir of bioactive compounds with broad antibacterial, antifungal, antiparasitic, and antiviral properties. In this study, we focused on monensin, a polyether ionophore produced by *S. cinnamonensis*, and brefeldin A, a fungal metabolite derived from *E. brefeldianum*. Both compounds have recently garnered attention for their potential antiviral properties. Monensin was reported to inhibit several human coronaviruses by acting post-entry and reducing viral RNA and protein expression in epithelial cells and organoid models [25]. Likewise, brefeldin A has been shown to impair replication of dengue virus 2 by disrupting vesicular trafficking and host secretory pathways [40].

Our data demonstrate that monensin and brefeldin A exhibit antiviral activity against TOSV at concentrations that show minimal cytotoxicity in Vero cells (Figure 2A,B) and in human erythrocytes (Figure 2C,D). Time-of-addition experiments revealed distinct temporal patterns of antiviral action (Figure 3A and Figure 4A). Monensin displayed a rapid onset of activity, with strong inhibition observed as early as 4 h post-exposure (IC_50_ = 2.7 µM), and substantial antiviral activity was maintained at 6 h (IC_50_ = 2.5 µM) and 12 h (IC_50_ = 4.9 µM). Its reduced efficacy at 24 h may reflect either metabolic inactivation, compensatory cellular mechanisms, or the timing of TOSV’s replication cycle.

In contrast, brefeldin A exhibited a delayed antiviral effect, becoming effective only after 12 h of exposure (IC_50_ = 66.9 nM). The absence of inhibition at earlier time points suggests that Brefeldin A interferes with later stages of TOSV replication, potentially during virion assembly or egress, which rely heavily on intact Golgi and secretory pathways. The evaluation of the SI is essential to determine the therapeutic window between the concentration required to achieve antiviral activity and the concentration that induces cytotoxicity. The calculated SI values for monensin and brefeldin A are reported in Table S1. Monensin exhibited higher SI values at 4 h hpi (SI = 16) and 6 hpi (SI = 13.8), whereas brefeldin A showed a lower SI, with the highest value observed at 12 hpi (SI = 5).

The antiviral results were corroborated at the molecular level by qPCR data (Figure 3B and Figure 4B), which showed that monensin suppressed *L* gene expression at all examined time points in a concentration-dependent manner, whereas brefeldin A exerted a significant effect only at 12 and 24 h. Together, these results suggest that the two metabolites act at distinct stages of the viral life cycle.

To further investigate the cellular mechanisms underlying their antiviral effects, we assessed the protein expression of TFE3, a transcription factor activated during Golgi stress (Figure 5). Monensin treatment caused a clear, concentration-dependent upregulation of TFE3 after 4 h (Figure 5A). This aligns with previous reports showing that ionophores disrupt Golgi homeostasis by altering intravesicular ion gradients, thereby activating TFE3-mediated stress pathways [41]. Brefeldin A, despite its strong ability to collapse the Golgi into the endoplasmic reticulum, induced only a modest increase in TFE3 levels (Figure 5B). This limited and delayed activation is consistent with previous studies indicating that brefeldin A engages the TFE3-Golgi stress pathway less robustly than other Golgi stressors, such as the steroidal saponin OSW-1 [42,43]. The weaker response may reflect differences like cellular perturbations induced by the two compounds: monensin affects ionic balance and vesicular pH, whereas brefeldin A primarily disrupts ARF-dependent trafficking. Interestingly, TOSV infection in the absence of treatment led to a marked reduction (58%) in TFE3 expression. This finding suggests that TOSV may actively suppress components of the Golgi stress response, potentially as an immune evasion strategy, similar to what has been described for other enveloped RNA viruses such as coronaviruses [44]. The ability of Monensin to counteract this suppression and restore TFE3 levels might contribute to its antiviral activity by creating an intracellular environment less favorable to viral replication. Taken together, our findings indicate that monensin and brefeldin A are capable of inhibiting TOSV replication at concentrations that maintain an acceptable safety profile, as reflected by cytotoxicity and SI evaluations. These results highlight the potential of host-targeted antiviral strategies to interfere with distinct stages of the viral life cycle, offering a framework for the development of novel therapeutic approaches against TOSV. While further preclinical and in vivo studies are required to assess pharmacokinetics, dosing, and safety in complex biological systems, our study provides a proof-of-concept that microbial metabolites can serve as a source of clinically relevant antivirals, which may also have broader implications for the management of emerging phleboviral infections in endemic regions.

## 5. Conclusions

In summary, this study provides the first evidence that monensin and brefeldin A exert antiviral activity against TOSV, though with distinct kinetic and mechanistic profiles. Monensin acts rapidly and consistently across all tested time points and is associated with robust activation of TFE3, suggesting that induction of Golgi stress may contribute to its antiviral mechanism. Brefeldin A exhibits delayed but potent antiviral activity, likely acting through disruption of late secretory pathways essential for TOSV maturation. These findings identify two microbial metabolites with promising antiviral properties and highlight the relevance of targeting cellular pathways, especially those related to Golgi dynamics, as a strategy against emerging arboviruses. Given the limited therapeutic options available for TOSV, monensin and brefeldin A may serve as starting points for developing new antiviral agents. Future studies should explore the potential of structural analogs or derivatives with improved safety profiles and the effects of these compounds in physiologically relevant models such as neuronal or endothelial cells, which are natural targets during TOSV infection. A deeper understanding of these mechanisms will provide valuable insights into host–virus interactions and may guide the discovery of broad-spectrum antivirals effective against phleboviruses and other arboviruses.

## Figures and Tables

**Table 1:** CC_50_ values of monensin and brefeldin A after 6 and 24 h of exposure on Vero cells.

Time	Monensin (µM)	Brefeldin A (nM)
6 h	34.5	478.3
24 h	34.1	242.6

**Table 2:** IC_50_ values of monensin after 4, 6, 12, and 24 h of exposure against TOSV infection.

Hours of Exposure	IC_50_ (µM)
4 h	2.7
6 h	2.5
12 h	4.9
24 h	12

**Table 3:** IC_50_ values of brefeldin A after 4, 6, 12, and 24 h of exposure against TOSV infection.

Hours of Exposure	IC_50_ (nM)
4 h	>175
6 h	>175
12 h	66.9
24 h	>175

## Data Availability

The data presented in this study are available on request from the corresponding author.

## Supplementary Materials

The following supporting information can be downloaded at: https://www.mdpi.com/article/10.3390/v18030287/s1, Table S1. CC_50_, IC_50_, and SI values of monensin and brefeldin A against TOSV.

## Abbreviations

The following abbreviations are used in this manuscript:

TOSV	Toscana virus
CNS	Central nervous system
HSV-1	Herpes simplex virus type 1
HSV-2	Herpes simplex virus type 2
HCMV	Human cytomegalovirus
DMEM	Dulbecco’s Modified Eagle Medium
h	Hours
FBS	Fetal Bovine Serum
MTT	3-(4,5-dimethylthiazol-2-yl)-2,5-diphenyltetrazolium bromide
DMSO	Dimethyl sulfoxide
CTRL+	Positive control
CTRL−	Negative control
MOI	Multiplicity of infection
CMC	Carboxymethylcellulose
qRT-PCR	Real-Time Quantitative PCR
Ct	Cycle threshold
GAPDH	Glyceraldehyde 3-phosphate dehydrogenase
SDS-PAGE	Sodium dodecyl sulfate-polyacrylamide gel electrophoresis
SD	Standard deviation
CC_50_	50% cytotoxic concentration
IC_50_	50% inhibitory concentration
SI	Selectivity index
