# Supplementary material for: Antiviral Activity of Microbial Metabolites Monensin and Brefeldin A Against Toscana Virus: In Vitro Evaluation and Mechanistic Insights
Source: Viruses. 2026 Feb 27;18(3):287. doi: 10.3390/v18030287 (PMC13030401; doi:10.3390/v18030287)
Supplement: Supplementary file 1 [file viruses-18-00287-s001.zip › viruses-4157453-supplementary.pdf]

Table S1. CC_{50}, IC_{50}, and SI values of monensin and brefeldin A against TOSV.

	Monensin (μM)			Brefeldin A (nM)		
**Time**	**CC_{50}**	**IC_{50}**	**SI**	**CC_{50}**	**IC_{50}**	**SI**
4 hours	43.4	2.7	16	686.3	>175	nd
6 hours	34.5	2.5	13.8	478.3	>175	nd
12 hours	34.4	4.9	7	335.3	66.9	5
24 hours	34.1	12	2.8	242.6	>175	nd

nd: not determined.
